# Tumor Necrosis Factor-*α*: The Next Marker of Stroke

**DOI:** 10.1155/2022/2395269

**Published:** 2022-02-27

**Authors:** Yimeng Xue, Xianwei Zeng, Wen-Jun Tu, Jizong Zhao

**Affiliations:** ^1^Department of Neurosurgery, Beijing Tiantan Hospital, Capital Medical University, Beijing, China; ^2^Savaid Medical School, University of Chinese Academy of Sciences, Beijing, China; ^3^Rehabilitation Hospital of the National Research Center for Rehabilitation Technical Aids, Beijing, China; ^4^Institute of Radiation Medicine, Chinese Academy of Medical Science & Peking Union Medical College, Tianjin, China; ^5^Center of Stroke, Beijing Institute for Brain Disorders, Beijing, China; ^6^Beijing Key Laboratory of Translational Medicine for Cerebrovascular Disease, Beijing, China; ^7^China National Clinical Research Center for Neurological Diseases, Beijing, China

## Abstract

Although there is no shortage of research on the markers for stroke, to our knowledge, there are no clear markers that can meet the needs of clinical prediction and treatment. The inflammatory cascade is a critical process that persists and functions throughout the stroke process, ultimately worsening stroke outcomes and increasing mortality. Numerous inflammatory factors, including tumor necrosis factor (TNF), are involved in this process. These inflammatory factors play a dual role during stroke, and their mechanisms are complex. As one of the representatives, TNF is the primary regulator of the immune system and plays an essential role in the spread of inflammation. In researches done over the last few years, tumor necrosis factor-alpha (TNF-*α*) has emerged as a potential marker for stroke because of its essential role in stroke. This review summarizes the latest research on TNF-*α* in stroke and explores its potential as a therapeutic target.

## 1. Introduction

Stroke is the leading cause of death and long-term disability worldwide, and its incidence is increasing at younger ages [1, 2]. The high mortality and disability rates place a severe burden on society [3, 4]. Thus, the search for biomarkers that can predict disease prognosis or targeted therapy is significant to improve the treatment and reduce the disability rate [5]. However, there are no specific markers that can provide predictive and therapeutic information as far as we know. Previous studies by Simats et al. have summarized the role of inflammatory biomarkers in helping predict outcomes in stroke patients which may even become therapeutic targets [6]. The inflammatory response process runs through the entire stroke course [7]. In this cascade of inflammatory changes, cytokines like interleukin (IL), TNF, and interferon (IFN) act as central mediators in the inflammatory cascade and are considered as a therapeutic target and prognostic biomarker [8]. The researchers observed changes in the concentrations of several types of these cytokines in the cerebrospinal fluid and blood of stroke patients, and these changes were associated with prognosis [9–12]. TNF-*α* is an emerging molecule that is a kind of pleiotropic cytokine as the primary regulatory factor of the immune system that can be produced by a variety of cell types and is involved in a wide range of pathological processes [13, 14]. It plays a homeostasis and pathophysiological role in the central nervous system. Under pathological conditions, microglia release large amounts of TNF-*α*, which is a crucial component of the neuroinflammatory response associated with various neurological diseases [15]. Based on several robust pieces of evidence, changes in TNF-*α* were associated with stroke injury and stroke recovery [16–18]. For example, Tuttolomondo et al. reported that TNF-*α* expression was elevated after stroke, which stimulated the expression of tissue factors and leukocyte adhesion molecules and inhibited the fibrinolytic system [19]. Although several studies have reported contrary results, the use of TNF-*α* as a marker of stroke remains promising. In addition to the role of TNF-*α* in stroke, anti-TNF-*α*-based antistroke therapies have received increasing attention from the researchers. In a preclinical study, TNF-*α* receptor inhibitors reduce brain damage by reducing inflammatory responses in a rat model of ischemic stroke [20]. Therefore, this article reviews the research progress of TNF-*α* and its antagonists and discusses its application prospect in the treatment of stroke.

## 2. TNF-*α* Molecule and Its Receptor

**Figure 1:**
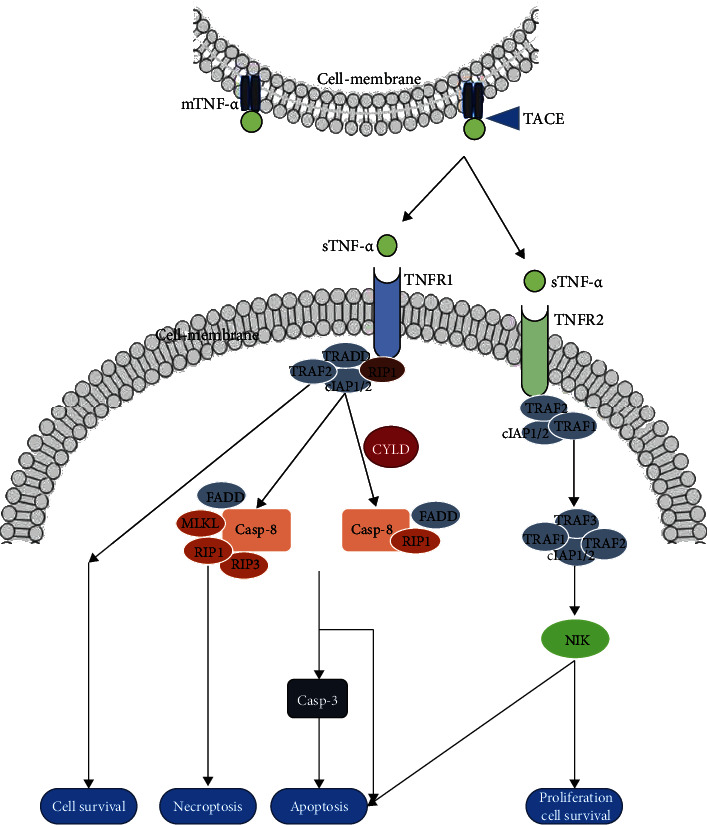
TNF-*α* binds to receptors and affects intracellular signal transduction. MTNF-*α* is hydrolyzed and cleaved by TACE to produce STNF-*α*. STNF-*α* binds to TNFR1 and TNFR2 through different signaling pathways, ultimately leading to a series of outcomes, including necrosis, apoptosis, survival, and proliferation.

TNF-*α* is produced by various cells, but its primary source is the cells of the immune system, such as macrophages, lymphoid cells, and mast cells [14, 21]. In these cells, TNF-*α* is first synthesized into transmembrane protein (tmTNF-*α*), which is then cleaved by matrix metalloproteinase TNF-*α*-converting enzyme (TACE) to release soluble TNF-*α* (sTNF-*α*) homotrimer and can bind to two types of receptors, namely, TNF receptor (TNFR) type 1 (TNFR1) and type 1 (TNFR2) [15, 21–23]. These two receptors are expressed differently in various cells and differ functionally [24]. Unlike TNFR1, which is ubiquitously expressed in all cell types, TNFR2 is expressed by some immune cells and preferentially by some Treg cells, some endothelial cells, and nerve tissue cells [13, 25]. The TNF-signaling complex structure enables TNF-*α* to induce inflammation and cell death or to induce tolerance to ischemia after stroke [26]. The main role of TNFR1 is to initiate apoptosis through its death domain and also to induce cell survival mechanisms [27]. Activation of the TNFR2 pathway by TNF-*α* contributes to immune response and inflammation [28]. It can affect the activation of many intracellular signaling pathways and ultimately lead to cell survival, cell migration, apoptosis, and necrosis (Figure 1) [29–31].

Although TNF-*α* has a higher affinity for TNFR2 than for TNFR1, most of the biological activities of TNF-*α* are initiated by TNFR1 [32]. The structure of TNFR1 includes a death domain (DD), which is constitutively expressed in most cell types and is activated by TNF-*α* in the form of membrane binding (mTNF-*α*) or soluble (sTNF-*α*) [33]. Activation of TNFR1 leads to trimer formation, which promotes DD recruitment of TNFR1-associated death domains (TRADD), and TRADD further recruits serine/threonine-protein kinase (RIPK) and TNFR-associated factor (TRAF) 2 [34–36]. The specific process can be described as TNFR1 binds to trimer TNF-*α* to release death domain silencer (SODD) protein. The TNFR-associated death domain (TRADD) binds to the TNFR1 death domain (DD) and recruits adapter protein receptor-interacting protein (RIP), TNFR-associated factor 2 (TRAF2), and Fas-associated death domain (FADD). When TNFR1 signals apoptosis, FADD binds to procaspase-8 and activates it, eventually initiating the protease cascade reaction. Activation of endonuclease (such as EndoG) mediates DNA breakage and leads to apoptosis. When TNFR1 signals survival, TRAF2 is recruited to the complex, inhibiting apoptosis by cytoplasmic apoptotic protein inhibitor (cIAP). Activation of TRAF2 results in activation of cFos/cJun transcription factors through mitogen-activated protein kinase (MAPK) and cJun N-terminal kinase (JNK) [37, 38]. The TNFR1 core signaling complex is thus formed and stabilized by RIPK1 ubiquitination, which ultimately mediates a cellular response. For example, cytokine signaling and cell survival are induced by activation of the NF-*κ*B, JNK, and p38 pathways [39, 40]. The apoptotic pathway would be activated in the absence of complete ubiquitination of RIPK1, leading to cell apoptosis or necrosis [41].

TNFR2 has no dead domain and is only fully activated by mTNF-*α* [42]. TNFR2 forms trimer and directly recruits TRAF2, TRAF1, or TRAF3 [43]. The nuclear factor kappa-light chain enhancer (NF-*κ*B), Akt (protein kinase B), and mitogen-activated protein kinase (MAPK) of B cells are then activated to initiate their biological function [44, 45]. For example, it promotes cell activation, migration, and proliferation; plays a protective role in cells; affects the amplification and function of Treg; and also mediates apoptosis through its cooperation with TNFR1 [45–47].

## 3. Physiological Role of TNF-*α* Molecule in the Central Nervous System

In the adult brain, TNF is mainly derived from glia, astrocytes, and microglia, and its levels are low, but its role in the central nervous system (CNS) is complex and multipotent [48–50]. First, TNF-*α* regulates normal neurotransmitter processes in different ways. For example, it not only can induce a rapid increase in AMPA receptors but also can decrease AMPAR levels in cortical surface and hippocampal neurons (a process achieved in the striatum through the elimination of Ca^2+^ permeability inhibition) and enhance tetrodotoxin insensitive Na^+^ channel currents in the plasma membrane of dorsal root ganglion (DRG) neurons. Furthermore, it also regulates the release of glutamate by astrocytes [51–57]. Second, TNF-*α* plays a dual role in neurogenesis through different inductive environments and receptor subtypes [58]. For example, TNF-*α* can cause progenitor cell death by abruptly stopping cell division [59]. It exerts neuroprotective effects when it binds to TNFR2 receptors expressed by human neural stem cells [60]. Third, TNF-*α* can affect endothelial cells in CNS. These pathways include influencing the morphology of endothelial cells, thereby affecting BBB permeability, enhancing the adhesion between leukocytes and endothelial cells, thereby facilitating leukocyte migration to the central nervous system and inducing angiogenic mediators that affect vascular endothelial cells proliferation [61–63].

## 4. TNF-*α* in Stroke

The etiology of vascular lesions is obviously redox reaction and stress-dependent [64]. In stroke, neurovascular units can become dysfunctional due to the lack of oxygen and nutrients [65]. During ischemia, changes in the brain include the release of glutamate, the production of reactive oxygen species (ROS) that cause oxidative stress, and activation of microglia, which can affect the secretion of proinflammatory mediators [66, 67]. Oxidative stress and inflammatory response have bidirectional effects on the whole stroke process. When blood vessels are occluded or underperfused, the immune response begins near the ischemic parenchyma and then extends to the ischemic zone, eventually spreading throughout the body, and microglia are activated and promote the release of TNF-*α* [68, 69]. Studies have shown that levels of TNF-*α* in brain tissue may continue to rise 1 day after ischemic injury and correlate with their severity [70, 71]. TNF-*α* is a core mediator in the immune processes of infection control, autoimmunity, allergic diseases, and antitumor activity [15]. The mechanism of TNF-*α*'s influence on vascular endothelium includes stimulating the expression of tissue factors and leukocyte adhesion molecules, activating matrix metalloproteinases, and producing oxidative stress through xanthine oxidase [61, 72]. These actions trigger local segments of blood vessels and lead to local inflammation, thrombosis, and bleeding [73]. Other studies have shown that TNF-*α* can disrupt the protective barrier between brain circulation. These effects include, first, stimulating the activation and proliferation of astrocytes and microglia and, second, regulating apoptosis factors, such as cysteine. Third, matrix metalloproteinase (MMP) transcription is induction in ischemia and penumbra inflammation. The last induced transcription of cytokines, such as IL-1 and IL-6 [74–77]. In addition, TNF can also induce ischemia tolerance and regulate the signal transduction of cerebral hypoxia and ischemia tolerance [78, 79]. In stroke outcomes, TNF-*α* is associated with epileptic seizures, movement disorders, spasms, aphasia, pain, depression, and cognitive impairment [80–83]. Zaremba et al. found that the level of TNF-*α* in cerebrospinal fluid (CSF) was significantly increased in stroke patients, and the increase of CSF and SERUM TNF-*α* in the first 24 hours of stroke was also significantly associated with the severity of a neurological stroke and the degree of dysfunction according to SSS and BI scores [84]. However, in a clinical study, the researchers found that the level of TNF-*α* was not associated with functional outcomes after acute stroke [85]. We speculate that this is because of how TNF-*α* plays a role in stroke prognosis, which is complex and diverse, and these specific mechanisms need to be further investigated. Doll et al. reviewed several preclinical and clinical studies suggesting that TNF-*α* has neurotoxic or neuroprotective effects in stroke. There were also conflicting findings when TNF-*α* was used to predict prognosis. These seem to indicate that the action of TNF-*α* is complex and bidirectional [26]. Because TNF-*α* ligand-receptor interactions are involved in almost every aspect of stroke-induced brain injury, it is a promising direction to use TNF-*α* as an inflammatory marker to predict the outcome of stroke. On the other hand, when TNF-*α* is used as a potential therapeutic target for stroke, blocking TNF-*α* can reduce focal ischemic injury and improve clinical outcomes [83, 86].

## 5. TNF-*α* Inhibitors

Ischemic stroke is a catastrophic disease. Unfortunately, because of the limited time window for treatment, only a small number of patients receive tissue plasminogen activator (tPA), which is the primary treatment; as a result, most patients receive only supportive care [6, 26]. It is urgent to renew the therapeutic drugs in the clinic. The positive effects of treatment targeting TNF-*α* in stroke have been demonstrated in preclinical studies over the past few years (Table 1). There are three effective ways to interfere with TNF-*α* action by blocking receptors, interfering with TNF-*α* signal transduction, and removing TNF-*α* protein in effectors [87]. Currently, TNF-*α* inhibitors, including enanercib, infliximab, adalimumab, pertuzumab, and golimumab, are mainly used to treat autoimmune diseases or inflammatory diseases [87–89]. Intraventricular injection of TNFR1 decoy receptors or anti-TNF-*α* antibodies, as well as systemic injection of TACE inhibitors, can reduce ischemic brain damage in stroke [90, 91]. After injecting TNF-*α* receptor inhibitor R-7050 into stroke rats, Lin et al. found that R-7050 reversed neuronal changes, TNF-*α* receptor/NF-*κ*B inflammatory signaling, and BBB destruction and ultimately reduced the area of cerebral infarction [20]. In another study, in older animals, mice treated with adalimumab (TNF-*α*-inhibiting antibody) reduced poststroke defects and improved poststroke survival [92]. When the preclinical experiment is transformed into clinical application, the researchers must overcome the adverse reactions. These include the most worrisome severe infections, malignancies, heart failure, and nerve demyelination, as well as other general side effects, such as headache, rash, anemia, pharyngitis, diarrhea, nausea, and abdominal pain [88, 93, 94]. Finally, the safety of anti-TNF-*α* agents during pregnancy or lactation needs to be further explored [88]. In the meantime, the researchers are still working to develop other types of inhibitors to improve stroke outcomes. For example, the IL-2/IL-2R antibody complex enhances Treg-induced neuroprotective effects by inhibiting TNF-*α* induced inflammation [95]. Contreras et al. proposed that the trimer TNF-R2 extracellular domain might be an innovative TNF-*α* antagonist [96]. Targeting P2X4 receptors improves postcentral stroke pain through the TNF-*α*/TNFR1/GABAAR pathway [97]. Given the fact that TNF-*α* inhibitors are less effective at penetrating BBB, the researchers are also looking for new types of inhibitors that can more easily move through BBB and act more effectively in the damaged areas [98]. These emerging studies provide new research ideas for anti-TNF-*α* treatment of stroke.

## 6. Conclusion

In conclusion, although some current studies do not support TNF-*α* as a clear marker of stroke, we still believe that it is desirable to focus on TNF-*α* in the following studies, considering that TNF-*α* is involved in the occurrence, development, and prognosis of stroke and has an indicative effect on the disease. Therefore, it is a promising research direction to use TNF-*α* as a biomarker of stroke development process or prognosis. At the same time, anti-TNF-*α* therapy can reduce brain damage in stroke, and it is also worth exploring as a therapeutic target. To make TNF-*α* be a reliable marker of stroke, the specific role and mechanism it plays in stroke, the protective effect and mechanism of anti-TNF-*α* treatment against brain injury, and how to reduce the side effects of antibodies are the primary issues that need to be further studied and solved by the researchers. There is a reason to believe that the next marker of stroke is on the horizon with the ongoing research.

## Figures and Tables

**Table 1:** Current research reports on use of TNF inhibitors in stroke.

Drug name	Drug type	Research type	Describe	Ref.	Year
R-7050	TNF-*α* receptor inhibitors	Preclinical	Using a rat model of permanent cerebral ischemia, pretreatment with R-7050 offered protection against poststroke neurological deficits, brain infarction, edema, oxidative stress, and caspase 3 activations.	[20]	2021
Adalimumab	TNF-*α*-neutralizing antibody	Preclinical	Older animals treated with adalimumab show a tendency to reduce poststroke defects and improve survival in older animals after stroke.	[92]	2021
Infliximab	TNF-*α* inhibitor	Preclinical	Improving stroke outcomes in a mouse model of rheumatoid arthritis.	[18]	2019
Alpha-lipoic acid and etanercept	Free radical scavenger/TNF-*α* inhibitor	Preclinical	By inhibiting peripheral TNF-*α* and downregulating microglia activation, it has protective effect on ischemic stroke rats.	[99]	2015
Infliximab and etanercept	TNF-*α* inhibitor	Preclinical	Compared with untreated rats, the volume of cerebral infarction was significantly reduced in the etanercept or infliximab group.	[86]	2015
Etanercept	TNF-*α* inhibitor	Preclinical	Decreased middle cerebral artery remodeling but increased cerebral ischemia injury in hypertensive rats.	[100]	2014
CNTO5048	TNF-*α* antibody	Preclinical	In a mouse model of intracerebral hemorrhage, posttraumatic treatment with CNTO5048 reduced neuroinflammation and improved functional outcomes.	[101]	2013
Etanercept	TNF-*α* inhibitor	Clinical	Perispinal administration of etanercept improves clinical symptoms in patients with chronic neurological dysfunction following stroke and traumatic brain injury.	[102]	2012
CTfRMAb-TNFR	Fusion protein	Preclinical	CTfRMAb-TNFR fusion protein treatment can reduce hemispheric, cortical, and subcortical stroke volume and neurological deficits and prevent stroke.	[103]	2012

## Data Availability

Please contact the corresponding author (Pro. Tu) for the data request.
